# The Importance of Connection to Others in QoL in MSA and PSP

**DOI:** 10.1155/2017/5283259

**Published:** 2017-09-28

**Authors:** Louise Wiblin, Rory Durcan, Mark Lee, Katie Brittain

**Affiliations:** ^1^Clinical Ageing Research Unit, Newcastle University, Newcastle upon Tyne NE4 5PL, UK; ^2^St Benedict's Hospice for Specialist Palliative Care, St. Benedict's Way, Sunderland SR2 0NY, UK; ^3^Department of Nursing, Midwifery & Health, Northumbria University, Coach Lane Campus West, Room B128, Newcastle upon Tyne NE7 7XA, UK

## Abstract

Multiple System Atrophy (MSA) and Progressive Supranuclear Palsy (PSP) are atypical Parkinsonian disorders with extended morbidity and reduced lifespan, known to have marked and early impact upon quality of life (QoL). This study aimed to address the lack of studies in the literature regarding personal perspectives on QoL in MSA and PSP in both patients and carers. Participants took part in qualitative, in-depth interviews in the North East of England, exploring what impacts their QoL and their experiences of living with these complex conditions. Connection to others was found to be a prevailing theme, encompassing difficulty communicating, social isolation, impact on personal relationships, and stigma. This work is helpful in that it emphasises the personal experiences of these patients and carers, which can provide insights into important areas for clinical service planning and best clinical management of individual patients as well as considerations for future research into QoL in these rare disorders.

## 1. Introduction

Multiple System Atrophy (MSA) and Progressive Supranuclear Palsy (PSP) are sporadic atypical Parkinsonian disorders (AP) which have poor response to symptomatic treatment, rapid and relentless progression, and reduced life expectancy compared with Parkinson's disease (PD) [1–3]. As these diseases have an especially aggressive course, in recent years, particular attention has been given to maximise quality of life (QoL) in these conditions, including the introduction of a palliative approach. Research looking at QoL specifically in MSA and PSP is lacking compared with the current body of work on QoL in PD [4–6]. QoL is frequently described as one of the key domains to improve for patients and caregivers in the context of Parkinson's disease and related disorders and the importance ascribed to QoL is growing [7]. However, a definitive measure which succinctly captures the essence of QoL has not been described; though many tools exist to try and capture QoL using quantitative scales, particularly in Parkinson's disease and conditions such as MSA and PSP [4, 8, 9]. Qualitative work to explore the “how” and the “why” of QoL and to gain an understanding from the patient and carer perspective has an important place in clinical research, particularly in QoL, as QoL is very much based upon an individual's perspective and reflection on the self. Qualitative methods are complementary to quantitative work, permitting access to the experiences of patients and carers that “other methods cannot reach” [10]. Qualitative work can act as a basis for developing clinical services or concepts for development of validated scales as well as reinforcing the important principles that lie at the heart of holistic medicine, understanding the patient's point of view and the diversity of experiences that patients and carers have [11, 12]. This study was carried out as part of a mixed methods project exploring QoL in MSA and PSP and a key finding in the qualitative portion of the investigation was the impact of connection to QoL in patients and in carers. Connection in this analysis refers to the way in which individuals are able to relate to others. This is via different means of communication, relating to people such as partners, family, or friends and how they felt others related to and perceived them. This has clinical implications, as good medical practice is built upon the development of patient and carer-relationships. Any barriers which patients and carers perceive should be identified wherever possible, to allow the best possible communication and rapport, as well as insight into their experience, and hence improve our management.

## 2. Methods and Ethics

This was an exploratory, qualitative project using semistructured interviews.

The study was approved by the Leeds-Bradford Research Ethics Committee. Participants were recruited from specialist atypical Parkinsonism clinics across three sites in the North East of England. All participants provided written informed consent. Participants were approached in clinic and provided with detailed information sheets informing them of the study aims, that participation was voluntary, that they could withdraw at any time, and that data would be treated confidentially. This qualitative study was part of a larger project exploring QoL in MSA and PSP using both quantitative and qualitative methods. Recruits to the project gave written consent if they wished to be approached to provide an interview. Any identifiable information was removed from transcripts, such as first names or surnames mentioned during interviews, to ensure the anonymity of interviewee and confidentiality was protected by the use of pseudonyms.

Patients had a diagnosis of MSA or PSP and carers were unpaid and voluntary. Purposive, pragmatic sampling was used to achieve a range and richness of experience with a balance of male, female, MSA, and PSP patients and a range of severities to give a more complete picture of living with AP. Particular effort was made to facilitate interviewing of participants with poor or negligible speech who used communication aids, as theirs is a poorly heard voice in clinical and research terms. In many articles in which interviews are performed with patients with Parkinsonian conditions, inability to communicate clearly is an exclusion criteria for participation, even in work on advanced disease. Indeed, few publications could be found by the authors (one included one patient with MSA and a mixture of neurodegenerative conditions and the other PD and stroke) describing the significant communication problems encountered in advanced neurological disease and allowing their participation [13, 14]. There was purposeful inclusion of these individuals into this qualitative study, permitting communication devices.

The interview schedule was produced with reference to the literature on MSA and PSP and QoL as well as qualitative work in PD (due to the lack of work looking at QoL and experience of living with MSA and PSP) [15–18]. It was developed by the interviewer (LW) with input from an experienced researcher in qualitative interviewing (KB). The schedule permitted broad coverage of key areas felt to be important after the literature review, encouraging a personalised response from each participant. The open questions of the interview guide could then be followed up by probes from the interviewer to explore elicited responses thoroughly. See Table 1.

**Figure 1:**
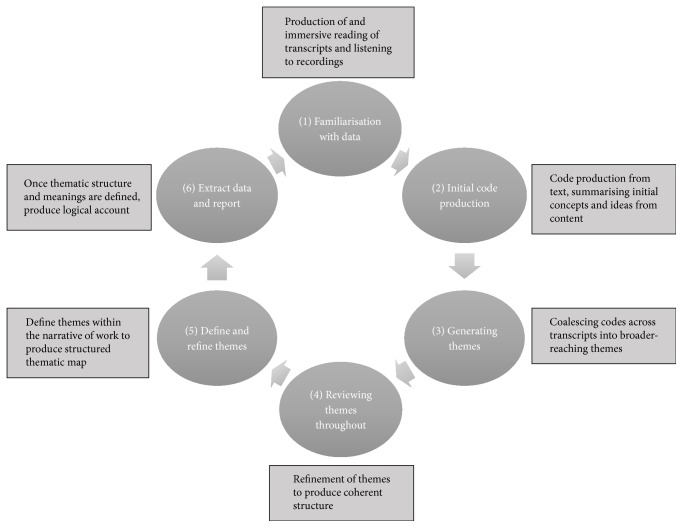
Stages of thematic analysis from Braun and Clarke 2006.

Interviews were carried out by LW as a one-to-one meeting either in a clinical research setting or in participant's homes, depending on which environment they were most comfortable in. Interviews were recorded on a digital device and transcribed verbatim. The interview transcripts were analysed using thematic analysis based upon the system described by Braun and Clarke [19]. See Figure 1.

In qualitative work, it is important in terms of transparency and rigour to describe the background of the researchers due to the influence this can have on their analysis. LW, the interviewer and primary researcher, is a training doctor in Neurology specialising in movement disorder with a particular interest in MSA and PSP and is an MD student looking at QoL and Palliative Care need in MSA and PSP. She undertook the main study design, participant recruitment, interviewing, analysis, and production of the manuscript. RD is a Geriatrics training doctor and movement disorder specialist who critiqued the analysis and contributed to the writing of the manuscript. ML is an advisor to the project and is a Consultant Physician and researcher in Palliative Care with an interest in movement disorder and was an advisor in the design of the project, the analysis process, and writing/review of the article. KB is an Associate Professor of Ageing & Health and is a Social Gerontologist. She advised on project design, reviewed and provided feedback on the coding process, and advised on project analysis and writing/review of the article.

QSR International NVIVO version 11 was used as an aid to analysis and data retrieval in thematic analysis. Coding was carried out as interviews were completed and were integrated into overarching themes; both data collection and analysis were an iterative process, whereby interviewing ceased when saturation took place, that is, when both LW and KB agreed that no more new, meaningful codes were being generated [20, 21].

Rigour was ensured by referring to qualitative guidelines such as COREQ and Yardley's criteria [22, 23]. See Table 2.

## 3. Results

Nineteen interviews were carried out in total, ten with patient participants and nine with carers. Four patients had MSA and there were four carers of individuals with MSA. There were six patients with PSP and five PSP-carers. Sixteen of the participants were patient-carer spouses and their relationships are described in Table 3.

**Figure 2:**
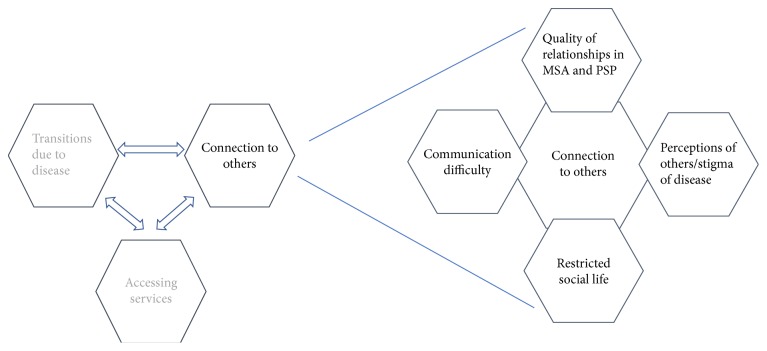
All prevailing themes from this project and their relationships to one another shown on the left. The theme “connection to others” and its associated subthemes are discussed in this article and are expanded on the right.

A prevailing theme which was found in analysis was that of “connection to others.” Other themes which emerged from the interviews using the topics covered in Table 1 were “transitions as a result of disease” and “accessing services.” This paper will explore the “connection to others” theme, as it is beyond the scope of the article to cover all three aspects. “Connection to others” was made up of several subthemes which will be described below and illustrated with extracts from interview transcripts; see Figure 2. Connection in this study refers to the ways in which the participants relate to others and ultimately how their ability to connect impacts their relationships with others. Relationships are intrinsically about connection, the definition being* “The way in which two or more people or things are connected, or the state of being connected*,*”* Oxford English Dictionary [24].

### 3.1. Communication Difficulty

In order to relate to others and have meaningful interactions, communication is vital. The patient participants in this study had variable speech difficulty. Some were profound, necessitating the use of electronic communication devices (one participant used an adapted iPad and another a light-writer), whilst some remained intelligible. However, the majority of patients found that their ability to communicate with others was impaired. A recurrent subtheme was patients finding reassurance that their speech or fluency was not severe, frustrating, as they felt their struggles to communicate were being dismissed.

Other people… I'll ask them, “Do I come across… ?” They always say, “Oh, you're fine.” I don't believe them, but they always say I come across fine.

(Doris, age 59, participant with MSA)

*The social life has deteriorated because I'm scared I might be a bit caught short or not being able to speak properly. You said earlier that my voice seemed okay. To me, it doesn't seem okay, I'm not as confident as I used to be. I couldn't sit down at a meeting anymore, a complete meeting, because I feel embarrassed losing my voice.*

(Gary, age 58, participant with PSP)

Gary, a participant with PSP, referred to a comment from LW prior to interview where the intent had been to encourage him that his voice would be clear when recording. This feedback is valuable in that it informs us that a social nicety of trying to give confidence to patients that their voice “isn't bad” may dismiss real concerns and distress which impact upon quality of life.

When the range of speech difficulty is considered, other participants were scarcely able to communicate verbally and needed to use electronic typing devices to “speak” for them.

  Interviewer:* do you think your main problem is… speech?*
  Sarah: ***speech***… ***yes***…
  Interviewer:* probably the speech*
  Sarah: ^*∗*^*typing sounds for 5 min*^*∗*^
  Interviewer:* I think I can read what*…
  Sarah:* mmmm*….

(Sarah, age 67, participant with PSP (bold text indicates “spoken” via light-writer device))

*First, I tried to put it down to the fact that she was doing this, but I don't think that is the case. I think she is starting to misspell words…and when it gets to, to, to press “Do”, sometimes she presses it, and it repeats and repeats and repeats. And I'd say, “No, the wrong- thing that's wrong is, you've pressed it too many times with your finger,” and I don't think she realizes….*

(Bob, 69, carer of Mary who has PSP)

Although these devices can enable people otherwise rendered unable to speak to make meaningful contact with others producing improvement in relationships and well-being as a consequence, the reality is that motor slowness, stiffness, and possible cognitive problems make communication devices increasingly difficult to use as the disease progresses. This can be frightening for patients and carers as they consider that their new “voice” may not be useable forever. This can be seen from Bob's statement above, as his wife Mary finds her light-writer more and more difficult to use and in Sarah who had profound slowness and rigidity, needing several minutes at a time to type short sentences or single words. This led to frustration where she tried to communicate with monosyllabic sounds when she was unable to type her thoughts.

### 3.2. Restricted Social Life

All participants in the study, across disease types, both patients and carers, described the negative impact of MSA or PSP on their ability to maintain a social life. This seemed to manifest in different ways. One frequent concern of patients with Parkinsonism is maintaining the volume of their voice. Work has suggested that patients with PD may have impaired ability to detect low volumes in their own voice, feeling that they are shouting when they are in fact difficult to hear. This likely adds to social awkwardness and feelings of effort or struggle in conversation [25, 26]. This can be seen by two patients with MSA, Doris and Rose, who particularly struggled to raise their voices and found themselves withdrawing socially as a consequence. This may lead to a profound change in perception of self and confidence.

I know my speech… nine times out of ten, I've got to repeat myself, and then I'll think, “Oh, I can't be bothered.” It's not worth it usually.

(Doris, age 59, participant with MSA)

*If I went to things, like the royalty dinners and things, there came to a certain point where really I couldn't take part in the conversation. Course you'd have a big round table. People were talking of course backwards and forwards, and they didn't, couldn't hear me. So I tended to just sort of sit back and just let things go on in front of me, and that was it, so I changed quite a bit. *

(Rose, age 71, participant with MSA)

Therefore, patients may be experiencing distress due to being less able to connect to others by speech, even if families and medical staff are not aware of any problem. The change may be innocuous, such as Rose's gradual shift during dinners to sitting back and letting the conversation flow around her as she realized her voice was becoming less able to cut through many voices in a loud social setting. This comparison with former selves and reducing abilities can produce a bereavement reaction for what has been lost.

From the carer's perspective, the burden of care and responsibility, especially the 24-hour nature of it, had an effect on social networks. Emma discussed the rare times she was able to meet with friends but found that she was unable to confide in them and she was afraid she would be unable to “click back” into a mindset which she uses to cope. This suggests that some barriers between carers and their old relationships and social lives grow because of their new responsibilities and experiences: perhaps not feeling that they share enough in common to confide in them. This produces a disconnect from society for the carer.

Unless they're very close to you… it's hard to click back into the “hi, yes how you today?” when the day before you broken down in tears over coffee, but I find that even, with my good friends which I think I mentioned to you, that you think twice about opening up to them. I hardly see any friends.

(Emma, age 61, carer of Matthew who has MSA)

What happens if I am not here, if she falls over?

(Earl, age 70, carer of Helen who has PSP)

*Because he has some horrendous falls…when he was at home before I went off for some respite he'd had- oh, he'd fallen and he was bruised. He looked like a car crash. He was always bumping into walls and stuff and falling back over and cracking his head. But I managed to keep him reasonably safe and intact as best I could.*

(Sally, age 70, carer of partner with PSP)

Earl and Sally discussed a very common subtheme in carers of both groups but most prominently in carers for people with PSP, the constant vigilance due to fear of their partner falling and injuring themselves. This seemed to encompass the full-time nature of caring and the emotional as well as physical pressures it exerts. This need to protect their spouse leads to an ever-shrinking social circle as their partner becomes less able to leave the home, resulting in social networks reduced to care-giver, patient, and occasional visitors to the home.

### 3.3. Perceptions of Others/Stigma of Disease

The ability to connect with others is influenced not only by the willingness and ability to communicate but also in how others perceive you (or possibly more importantly, how you believe others perceive you). Patients with MSA and PSP perceived stigma from others on the basis of their ability to interact. This was largely based on fear of perception from other from conversation, such as Gary feeling that his impaired fluency caused others to dismiss him or Rose's concern that her motor slowing would be seen as cognitive decline.

  Gary:* Yes. I feel as it's difficult. The others will take what I say and they'll understand it, but then they'll question it.*
  LW:* What do you mean, Gary?*
  Gary:* It's the way you say things to people. Words get jumbled up. They'll then say they understood me, but they didn't*.

(Gary, age 58, participant with PSP)

*…you have people sort of waiting whilst I slowly spoke to them. I didn't want them to think that I'd sort of- I think everybody thinks when you've got that and you slow down it might be a mental thing, which obviously it isn't because inside your head, it's all, it's really going on.*

(Rose, age 71, participant with MSA)

Mary, who used a light-writer to communicate, felt her inability to speak and the time she needed (due to a combination of bradykinesia and bradyphrenia) to type her responses caused her to believe herself judged by those around her. She felt that her combination of disabilities led to others believing that she was stupid and ignoring her, a powerful and profound insight into how these patients' symptoms impact upon their identity and well-being.

  Mary: ^*∗*^*typing sounds*^*∗*^ ***I sometimes think***  ^*∗*^*typing sounds*^*∗*^ …***people don't understand***
  LW:* what do people not understand that you want them to, can you pin that down?*…
  Mary: ***that I'm not stupid***
  Mary: ^*∗*^*typing sounds*^*∗*^ ***it's very frustrating***
  LW:* how do people react to your speech as it is?*
  Mary: ^*∗*^*typing sounds*^*∗*^ ***most people just ignore me***
  LW:* ignore you?*
  Mary: ***yes.***

(Mary, age 69, participant with PSP (bold text indicates “spoken” via light-writer device))

### 3.4. Quality of Relationships in MSA and PSP

Relationships and how they succeed or fail beyond a diagnosis of AP were a frequent theme. Relationships with others were frequently discussed within interviews. These relationships did not only include that between patient and carer (in this study, spouses) but also with other family members and friends.

As communication is so fundamental to relationships, the ability to have meaningful interactions with a partner is very important in maintaining that relationship through a diagnosis of a Parkinsonian disorder [27, 28]. This can be seen with Jackie, a carer who feels he can still communicate meaningfully with his wife Rose and with Sally, whose spouse is less able to speak (and was not able to give an interview) but she feels they are still able to communicate with each other.

Well deep down, no, it doesn't matter at all. We can have a nice night in together and we can communicate with one another. [Rose] loves talking although she has difficulty talking now and I have difficulty hearing her. She is a little bit deaf, you might have noticed that but I think quality of life, it is defining quality of life. When you get to real hardcore values, they're probably undiminished in my view but you have to be single minded to be able to identify that and I count myself fortunate.

(Jackie, age 73, carer of Rose who has MSA)

*Came back on the Sunday, I went straight to see [my husband]; tell him all about it and blah, blah, blah. He's happy to hear…we have quality time. We have meals together. I take him out; we go for a beer. I'm falling in love with him again.*

(Sally, age 70, carer of husband with PSP)

Julia, a participant with MSA, spoke positively of the impact her marriage and the relationships with her family had on maintaining home life with a degenerative disease. The use of the term “rock” is an interesting one, suggesting her husband keeps her tethered or connected despite her illness.

Exactly. Which is why I always refer to him as my rock. Because he is a stable influence in my life. He is just so down-to-earth, his feet are just so firmly cranked on the ground. He keeps saying, “That's no bother. That's no bother.”

(Julia, age 62, participant with MSA)

*The relationship [changing]? No, not really. Still love each other.*

(Tiberius, age 66, carer of Julia who has MSA)

Similarly, Julia's husband Tiberius, as he simply put it, did not feel that the quality of his relationship with his wife had changed, despite the change in her health and abilities and that they still loved each other. This support for people with chronic conditions is very valuable and may trigger medical and social teams to consider patients and carers more as a pair, supporting both, as the well-being of one is so fundamental to the other.

Interviewed participants discussed the difficulties in maintaining friendships which seemed to be multifactorial. The nature of friendship and even the friends themselves seemed to shift or change with diagnosis and increasing symptoms. Bryce, a participant with PSP, felt that certain friends were only interested in socializing when he was well enough to do so and lost interest in him when he became less physically able. From the statement below he feels discarded by his former peers.

  Bryce:* No, because- well, your friends yes, used to be. When you were all right your friends used to come around and see you. Since I've took bad I never see them.*
  LW:* Do you know why?*
  Bryce:* Yes, because they don't care. That's my opinion like.*

(Bryce, age 76, participant with PSP)

Bryce was the only participant within the cohort who did not have a partner or spouse. Therefore, the impact of this loss of interaction with friends was especially profound for him. Friendship is often built upon mutual interests, and being less able to participate may mean less contact with friends as those activities become less accessible.

## 4. Discussion

This qualitative study suggests that maintaining connection and appreciating the possible barriers that MSA and PSP patients and carers face in this challenge are vital in enhancing QoL. There is evidence in the literature that connection to others is key to QoL in other chronic, life-limiting illnesses [15]. This study has shown that, in a cohort of individuals with MSA and PSP and their carers, connection was a prevailing concern, with subthemes of affected social life, communication impairment, perception of stigma, and impact on relationships. In neurological disorders, it has been shown that individuals who have wider social networks and better support tend to have better outcomes [30]. Social isolation has been found to be a predictor for further ischaemic events after stroke [31]. Although many studies apprise QoL using HR-QoL (Health-Related QoL) tools which focus on well-being within the scope of illness and disability, qualitative work in PD and stroke has shown that participants' concerns regarding QoL are frequently related to maintaining connection to others such as spouses or family and shrinking social lives [15, 32]. Northcott and Hilari [33] described how stroke affected friendships with particular impact if there was pronounced speech disturbance; this led to isolation from friends due to embarrassment on the patient's part or desertion from the friends as they found the stroke survivor more difficult to communicate with. Friendships were particularly imperiled if they were based upon activities which patients become too disabled to take part in anymore, such as sport. These factors are particularly applicable to AP and were described by participants with the added implication of progressing, worsening symptoms, unlike stroke in which the deficit is fixed and will stay stable or even improve.

Being able to communicate, verbally or otherwise, is integral to being able to connect to others, individuals, and communities. It has been shown that people with PD feel excluded from conversations as speech becomes more effortful, impacting upon their relationships, personal identity, and dignity; they, like some participants in this study, felt that their voices were impaired even if others said they were not. These perceived communication problems were correlated with depression [34, 35]. Efforts to reconnect individuals with speech problems due to neurodegenerative disease can be life-changing and increase QoL, such as providing electronic communication devices in MND [36]. However, progressing motor and cognitive problems in MSA and PSP (and indeed, in MND) can make these aids more challenging to use as the disease progresses, as the two participants who used light-writers demonstrated in this work. Other practical measures we can take as medical practitioners include education, so clinicians, nurses, and therapists are aware of how these and similar conditions impede conversation, giving time and being creative in their approach in how to make connections to these patients. Simple signing, picture boards, and one-to-one sessions with trained specialists or volunteers may enable patients to relate their concerns, have these acknowledged, and improve QoL.

Stigma has been described in PD patients not just from physical changes in appearance but from decline in the ability to speak clearly and fluently [37]. It must also be recalled that stigma can be felt or enacted by others, but often the effect upon the person feeling this stigma is the same. PD patients in a review of a number of studies experienced impact on the quality of their interactions with others, producing a retreat from the social sphere [38]. Stigma has been found to predict negatively for QoL in PD [39]. When it is considered that speech problems in MSA and PSP are more severe and have an earlier onset and the conditions are overall less responsive to medication, these factors are especially important to consider in AP [40].

Finally, AP affects the care-giver's ability to connect to others. This is borne of the increasing support their relative needs, both physical and emotional, and the constant burden of vigilance and worry in keeping the patient safe. This can cut carers off from outside social life from the demands on their time and limited freedom [41]. This is seen in PD, though the rapidity and extended morbidity in AP would be likely to put more pressure on this carer group. Falls produce especial distress in carers, leading to fears of leaving the patient alone for any length of time, which has also been seen in PD, but again issues like motor recklessness in PSP may lead to these problems being more acute in AP [42].

## 5. Conclusions and Future Directions

This study is qualitative and based upon a group of participants in the North East of England; it is therefore not possible to conclude that its findings are generalisable. However, the strengths of this work include the subjective and personal insight into this group which is lacking in the literature; this is particularly meaningful when it is considered that QoL has a very personal and self-reflective element to it which may not be fully captured by quantitative survey data collection. Future work should consider QoL of patients and carers with AP, taking into account communication and relationships. The aim of this paper was to explore personal perspectives on QoL and how disease impacting connection to others can give clinicians insight into how AP affects patients and carers beyond a simple description of symptoms. The implications for practice are to emphasise the patient's need to communicate and feel connected to others, their carer, families, and friends and to feel they are involved in clinical decision-making. This is of paramount importance in maintaining QoL and allowing holistic assessment in these conditions for which we, as yet, have no curative treatment. Acknowledging the impact of speech issues on the QoL of patients and carers is key, giving time for patients to try and convey their concerns (even when very slow or needing many attempts to do so) and good, early speech and language input to maximise their ability to communicate by whatever means they have available. These findings also have wider societal implications. It is difficult for medical services to address these far-ranging issues of a disconnect from others in patients and carers alone. In the future, incorporating social care, the volunteer sector and even technological advancements to enhance communication and independence should complement medical services. QoL is often assessed using quantitative measures and whilst this is valuable, work with patient groups using qualitative methods has the potential to shine a light on concerns that we may never have known existed.

## Figures and Tables

**Table 1:** Basic semistructured interview schedule. Structure derived from Patton [29].

Semistructured interview topics
Background of life before diagnosis
Process of diagnosis
How have things changed
Discussion of relationships
How is MSA/PSP challenging
Experiences with medical teams
What is your understanding of Palliative Care

**Table 2:** Two criteria used to demonstrate quality and validity in qualitative work and how they apply to this study. From Tong et al. [22] and Yardley [23].

COREQ domain checklist summary	Yardley's qualitative validity criteria
Sampling method: purposive, pragmatic sampling	Sensitivity: study designed with respect to known literature with respect to patient input (patient/carer group consulted in design)

Setting: participant's homes or clinical research facility (choice given). One-to-one interviewing	Commitment and rigour: LW experienced in movement disorder and specialising in AP. Extensive review of literature, training in interviewing methods, and analysis overseen by KB, an academic with many years of experience in qualitative research

Method: thematic analysis. NVivo v. 11.0 used as analysis aid	Transparency: methods described in methods section, process used shown in Figure 1. Disclosure of researcher background and assumptions given

How was data recorded: recorded on digital device and transcribed verbatim	Impact and importance: having implications for planning of future services for AP and for improving best practice. Potential to impact QoL in a rare, underresearched group of conditions. Demonstrating the need for more work in the future

Description of themes: themes derived from data, not preselected then imposed	

Supporting extracts: quotations used throughout report	

**Table 3:** Participant demographics and relationships. *∗* indicates these participants communicated with an electronic device.

Pseudonym	Sex	Condition	Role	Age	Profession	Marital status
Matthew (MP1)	Male	MSA	Patient	64	Retired lawyer	Married to Sally
Emma (CM1)	Female	MSA	Carer	61	Retired charity worker	Married to Matthew
Sally (CP1)	Female	PSP	Carer	70	Retired dental nurse	Married
Bryce (PP3)	Male	PSP	Patient	76	Retired technician	Single
Doris (MP2)	Female	MSA	Patient	59	Retired librarian	Married to Bill
Bill (CM2)	Male	MSA	Carer	57	Director	Married to Doris
Rose (MP3)	Female	MSA	Patient	71	Retired teacher	Married to Jackie
Jackie (CM3)	Male	MSA	Carer	73	Retired head teacher	Married to Rose
Julia (MP7)	Female	MSA	Patient	62	Retired hotelier	Married to Tiberius
Tiberius (CM7)	Male	MSA	Carer	66	Retired hotelier	Married to Julia
Sarah^*∗*^ (PP4)	Female	PSP	Patient	67	Retired teacher	Married to Tom
Tom (CP3)	Male	PSP	Carer	70	Retired oil chemist	Married to Sarah
Helen (PP18)	Female	PSP	Patient	68	Retired newsagent	Married to Earl
Earl (Cp17)	Male	PSP	Carer	70	Retired chartered accountant	Married to Helen
Mary^*∗*^ (PP24)	Female	PSP	Patient	69	Retired newsagent	Married to Bob
Bob (CP23)	Male	PSP	Carer	69	Retired newsagent	Married to Mary
Gary (PP20)	Male	PSP	Patient	58	Retired project manager	Married to Pat
Pat (CP19)	Female	PSP	Carer	62	Analyst	Married to Gary
Jack (PP19)	Male	PSP	Patient	71	Retired HGV manager	Married
